# Evaluation of Knowledge, Attitudes, and Practices about Pharmacovigilance among Community Pharmacists in Qassim, Saudi Arabia

**DOI:** 10.3390/ijerph20043548

**Published:** 2023-02-17

**Authors:** Suhaj Abdulsalim, Maryam Farooqui, Mohammed Salem Alshammari, Meshal Alotaibi, Abdulfattah Alhazmi, Abdulmajeed Alqasomi, Waleed Mohammad Altowayan

**Affiliations:** 1 Department of Pharmacy Practice, Unaizah College of Pharmacy, Qassim University, Buraydah 52571, Saudi Arabia; 2 Department of Pharmacy Practice, College of Pharmacy, University of Hafr Al Batin, Hafr Al Batin 39524, Saudi Arabia; 3 Clinical Pharmacy Department, College of Pharmacy, Umm Al-Qura University, Makkah 24381, Saudi Arabia; 4 Department of Pharmacy Practice, College of Pharmacy, Qassim University, Buraydah 52471, Saudi Arabia

**Keywords:** adverse drug reaction reporting, community pharmacists, pharmacovigilance, Saudi Arabia

## Abstract

Background: Pharmacovigilance (PV) is an essential activity to detect adverse drug reactions (ADRs) and ensure patient safety. Hence, we aimed to evaluate knowledge, attitudes, and practices (KAP) regarding PV among community pharmacists in Qassim, Saudi Arabia. Methods: A cross-sectional study was conducted by using a validated questionnaire after obtaining ethical approval from the Deanship of Scientific Research, Qassim University. The sample size was calculated based on the total number of pharmacists in the Qassim area by using Raosoft, Inc. Statistical Package for the Social Sciences version 20 was used for data entry and analysis. Ordinal logistic regression was performed to identify the predictors of KAP. A *p*-value of <0.05 was considered statistically significant. Results: A total of 209 community pharmacists participated in the study; 62.9% of them defined the PV correctly, and 59% of them defined ADRs correctly. However, only 17.2% knew where to report ADRs. Interestingly, the majority of participants (92.9%) reported that it is necessary to report ADRs, and 73.8% of them were willing to report ADRs. A total of 53.8% of the participants identified ADRs during their careers; however, only 21.9% reported ADRs. Barriers discourage ADR reporting; the majority of the participants (85.6%) do not know how to report ADRs. Conclusion: Community pharmacists who participated in the study were knowledgeable about PV, and their attitude towards reporting ADRs was highly positive. However, the number of reported ADRs was low because of the lack of knowledge on how and where to report ADRs. Continuous education and motivation about ADRs reporting and PV are warranted among community pharmacists for the rational use of medications.

## 1. Introduction

Pharmacovigilance (PV) is defined as being aware of the activities related to the detection, evaluation, understanding, and prevention of the harmful effects of medications [1]. In 1961, international drug monitoring programs were established in collaboration with the World Health Organization (WHO), and in 2022, 153 countries were part of WHO programs [2]. PV aims to increase patient health and safety and provide information on the risks and benefits of medicines [3].

Adverse drug reactions (ADRs) represent a significant global clinical issue, causing substantial morbidity and mortality. The WHO defines ADR as “any noxious, unwitting, and unwanted effect of a drug, that appears in the doses in humans for prophylaxis, diagnosis, or therapy” [4]. Fatal ADRs registered in a WHO PV database, during the last 10 years (1 January 2010 to 31 December 2019), reported 43,685 fatal ADRs out of 3,250,967 ADRs registered, which corresponds to just over 1% of the total number of ADRs [5]. The safety and quality of life can be affected by ADRs, and there is a need to consider the cost to manage the ADRs [6].

ADRs are identified, not always in premarketing clinical trials, by continuously monitoring the safety of drugs to establish risk–benefit profiles. The spontaneous reporting system is a method used by healthcare professionals (HCPs) to report detected ADRs to the health authorities. It helps to find past undetected effects of a drug throughout the life of the drug but has limitations such as underreporting. Spontaneous reporting is the most essential part of the PV system. The spontaneous reporting system is a passive method that relies on HCPs reporting suspected ADRs. In Saudi Arabia, there is already a system in place that has been supported by the Saudi Food and Drug Authority (SFDA) [7]. The main goal of the SFDA is to control how safely and effectively pharmaceuticals, medical equipment, food, and even cosmetics are regulated in the kingdom. However, many HCPs are not aware of the importance of reporting ADRs. HCPs can work widely in reinforcing knowledge about the safety profile of a drug [8]. HCPs such as pharmacists are important to increase the safe and effective use of medications [9]. Organizations should direct exhaustive medication security and PV review to assess their compliance with worldwide laws, regulations, and guidance [8].

In the 21st century, the use of medications is increasing along with problems associated with medication use, warranting urgent attention from policymakers. Consequently, it is essential to guarantee the safe and effective use of medications. Moreover, continuous and timely efforts from various partners or stakeholders are necessary to assure the rational use of medications. Stakeholders include pharmaceutical manufacturers, government drug administrative specialists (e.g., the SFDA), and healthcare workers (doctors, drug specialists, medical caretakers, paramedical staff, etc.). In Saudi Arabia, the PV system is a relatively new concept because it has been initiated recently with the efforts of the SFDA, and interestingly, there were a huge number of reports observed in a short time [4]. However, a study from Madina-al-Munwara, Saudi Arabia, reported that a lack of knowledge about ADR reporting among HCPs highlighted the urgent need to carry out appropriate training for HCPs to improve the practice of ADR reporting [10].

The traditional role of pharmacists is limited to preparing and dispensing the medications prescribed by the physician. Recently, the pharmacists’ roles have evolved from a product-oriented approach to a patient-oriented approach. These roles include monitoring and reporting new ADRs and improving patients’ health and economic outcomes [11]. Being the most accessible healthcare professionals, community pharmacists need to have good knowledge, attitudes, and practices (KAP) in reporting ADRs [12]. The development of an awareness of medication safety and a culture of reporting ADRs in community pharmacists will improve medication safety.

In 2018, an e-prescribing program called WASFATY was introduced in Saudi Arabia, which in turn offered patients the ability to have their medications dispensed, initiated by physicians, and sent electronically to community pharmacies [13]. This in turn increases patient turnover, resulting in community pharmacies demanding a more aggressive role in ADR detection and reporting. The Qassim region of Saudi Arabia is densely populated, with an area of 65,000 km2 and with an approximate population of 1,016,756 [14]. The region has well-established primary and tertiary hospitals offering a wide range of clinical and therapeutic services. In addition, the Qassim region has well-established, privately operated community pharmacies that provide a broad variety of pharmaceutical and health-related services. Since the introduction of WASFATY in Qassim, this is the first study to focus on KAP and PV among community pharmacists in the Qassim region. Although there are few studies reported from Saudi Arabia about KAP of PV, very few data were available from the Qassim region. Hence, we took up this study to explore community pharmacists’ KAP regarding PV and assess the barriers to reporting ADRs.

## 2. Materials and Methods

### 2.1. Study Design, Criteria, and Settings

A cross-sectional study was conducted by using a validated questionnaire among community pharmacists in Qassim, Saudi Arabia. Hospital pharmacists and community pharmacists from outside Qassim were not included in the study. The study was conducted from September 2020 to April 2021.

### 2.2. Study Instrument

A validated and reliable KAP questionnaire was adopted with permission from the original authors [15]. The adapted questionnaire was modified based on the comments from four subject experts, including academicians and pharmacy managers, and piloting was performed on ten community pharmacists. A total of five sections were included in the final questionnaire (See Supplementary Materials). PV and ADR awareness was documented by five questions in the first section. Six questions were used in the second section of the questionnaire to assess perceptions and attitudes towards reporting ADRs. There were three questions in the third section of the questionnaire regarding the reporting of an identified ADR. The fourth part of the questionnaire included two open-ended questions to investigate the barriers in Saudi Arabia to establishing a PV center and any suggestions or recommendations. In the last section of the questionnaire, pharmacist demographics were included. The English version of the questionnaire was distributed among community pharmacists.

### 2.3. Sample Size

The sample size was calculated by using Raosoft^®^ (Sample Size Calculator; Raosoft inc.) [16], considering 5% as the margin of error and 95% as the confidence level, with a 50% response rate; the population considered was 500 community pharmacists. The sample size was 218 community pharmacists.

### 2.4. Data Analysis

Statistical analyses (data screening, descriptive and inferential analysis) were performed using SPSS version 20.0. In this study, all numerical data were presented by median and IQR as the data are not normally distributed. All categorical data were presented by count and percentage. Ordinal logistic regression analysis was performed to identify the predictors of KAP. In the logistic regression, age, gender, last degree, years of experience, country of graduation, and rank of employment were included as study parameters. A *p*-value of <0.05 was considered statistically significant.

## 3. Results

### 3.1. Demographic Details about Study Participants

Out of 218 questionnaires distributed 209 (96%) pharmacists completed the questionnaires. The median age of the study population was 32 years and most were Egyptian (n = 130, 62%) males (n = 193, 92%). The majority (n = 152, 72%) held a bachelor’s degree and the median practice years was 7. A total of 72 (34%) were from Unaizah city in the Qassim region. Additional demographic details are provided in Table 1.

### 3.2. Knowledge about PV and ADRs Reporting

Most participants (n = 130, 62%) selected the correct answer for the WHO definition of PV and ADR. When asked about the purpose of PV, 144 (69%) selected the correct answer. More than half (n = 118, 57%) of the participants preferred to send ADR information via e-mail or website, and 56 (27%) preferred direct contact. The details on knowledge about PV and ADRs are provided in Table 2.

### 3.3. Community Pharmacists’ Attitudes towards PV and ADR Reporting

Out of 209 pharmacists, 195 (93%) believed that ADR reporting was necessary; 172, (82%) pharmacists agreed that reporting ADRs is a professional obligation. Nearly all (n = 196, 94%) pharmacists believed that ADR reporting affects the health system positively. Most (n = 167, 80%) agreed that reviewing drugs can prevent harmful drug reactions; however, 26 (12%) answered “I don’t know”. A total of 183 (88%) pharmacists agreed that ADRs should be reported in healthcare settings. A total of 155 (74%) pharmacists were willing to report ADRs in their practice; 33 (16%) were not willing to report ADRs. Table 3 shows the details of the attitudes towards PV and ADR reporting.

### 3.4. Practices towards PV and ADR Reporting

Of the 209 participating pharmacists, 142 (68%) conducted medication reviews with patients. Moreover, 114 (55%) pharmacists had identified an ADR. However, only 46 (22%) pharmacists reported an ADR to the SFDA. Most (n = 137, 66%) agree that ADRs should be reported to the SFDA. Table 4 shows the details of practices towards PV.

### 3.5. Barriers Limiting the PV and ADR Reporting

Out of 209 participants, the lack of a formal process in place (n = 131, 63%) represents a major barrier. Other reported barriers were a lack of knowledge by the patient about their medication (n = 119, 57%), lack of time (n = 86, 41%), lack of training on how to conduct a medication review (n = 84, 40%), and communication difficulties (n = 56, 27%). Barriers towards PV and ADR reporting details are given in Table 5.

### 3.6. Factors Discouraging the PV and ADR Reporting

The most cited factor discouraging ADR reporting was not knowing how to report an ADR (n = 123, 59%). Regarding barriers to Saudi Arabia having a formal PV center, lack of training (n = 23, 11%) followed by lack of staff (n = 13, 6%) were the most cited reasons. Factors discouraging PV and ADR reporting details are provided in Table 6.

### 3.7. Regression Analysis

**Figure 1:**
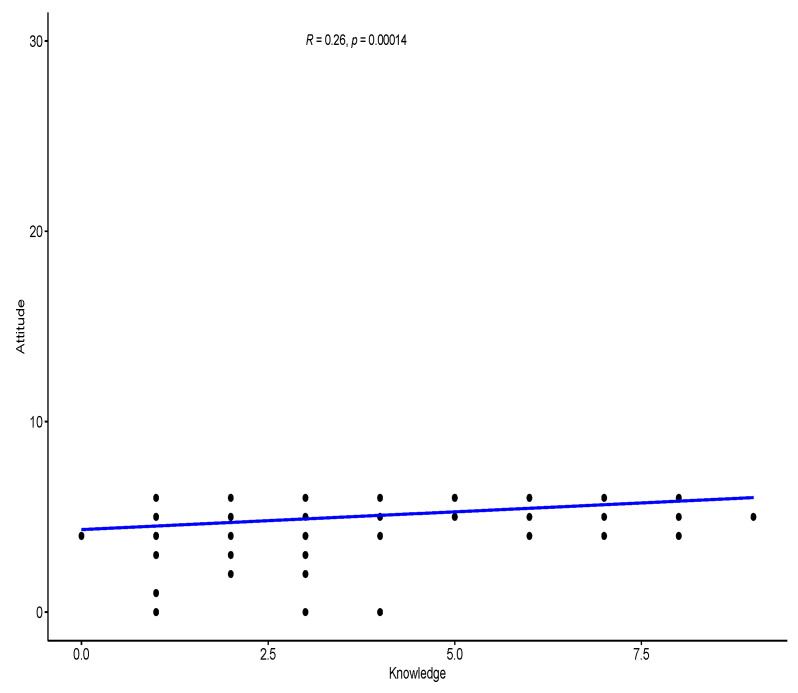
Scatter plot with regression line to show the correlation between knowledge and attitude. There is a statistically significant positive weak correlation between knowledge and attitude (R = 0.26).

**Figure 2:**
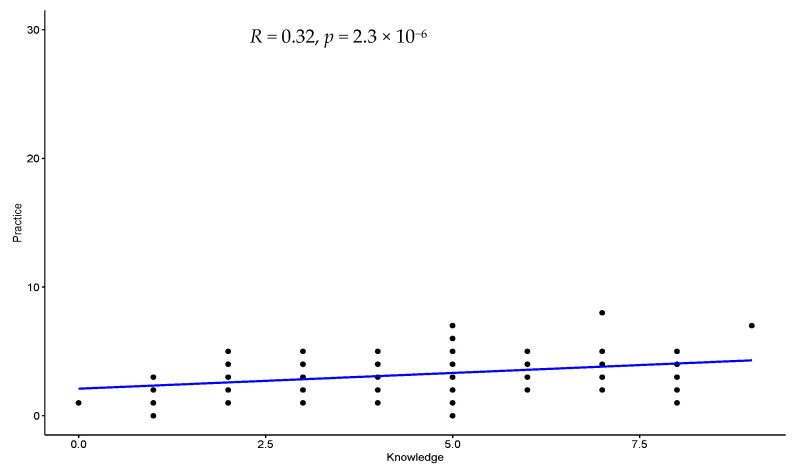
Scatter plot with regression line to show the correlation between knowledge and practice. There is a statistically significant positive weak correlation between knowledge and practice (R = 0.32).

**Figure 3:**
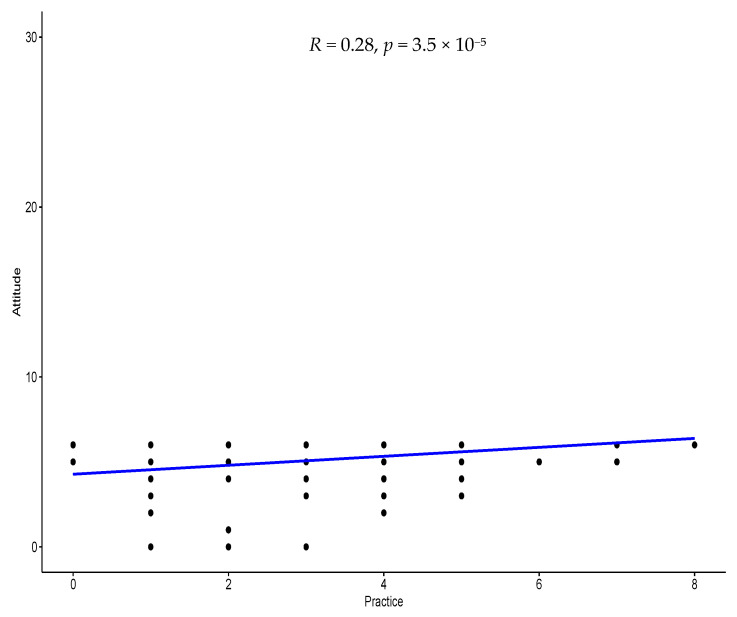
Scatter plot with regression line to show the correlation between attitude and practice. There is a statistically significant positive weak correlation between attitude and practice (R = 0.28).

The regression analysis with scatter plot showed a statistically significant positive weak correlation between knowledge and attitude (R = 0.26), knowledge and practice (R = 0.32), and attitude and practice (R = 0.28). Figure 1, Figure 2 and Figure 3 show the regression analysis with scattered plot.

### 3.8. Multiple Ordinal Regression to Show the Predictors of the Three Outcomes (Knowledge, Attitudes, and Practices)—Table 7

(1) Age

Each year of increase in age entailed a 3% decrease in the odds of having moderate or high knowledge in comparison with low knowledge, a nonsignificant 9% increase in the odds of having a moderate or high score in terms of attitude, and a 2% increase in practices from low to moderate or high practice.

(2) Gender

Being female increases the odds of having high or moderate knowledge by 24%, nonsignificantly increases the odds of having a moderate or high score in terms of attitude by 2.24, and increases the odds of moderate or high practice by 80% in comparison with males.

(3) Years of experience

With each year of experience, the odds of having moderate or high knowledge increases by 42%, the odds of having a moderate or high score in terms of attitude nonsignificantly decreases by 71%, and the odds of having moderate or high practice will decrease by 37%.

(4) Last degree (dichotomized into bachelor’s degree or higher degree)

By having a higher degree (M.Sc., Pharm.D., Ph.D.), the odds of having moderate or high knowledge increases by 3.56, the odds of having a moderate or high score in terms of attitude nonsignificantly increases by 5.04, and the odds of having moderate or high practice increases by 58% when compared to those who have a bachelor’s degree.

(5) Country of graduation (labeled as Saudi Arabia, Egypt, and others)

Graduation from Saudi Arabia decreases the odds of possessing moderate or high knowledge by 53%, nonsignificantly decreases the odds of having a moderate or high score in terms of attitude by 71%, and decreases the odds of moderate and high practice by 42% compared to those who graduate from Egypt.

Graduation from a country other than Saudi Arabia increases the odds of moderate or high knowledge by 70%, nonsignificantly decreases the odds of having a moderate or high score in terms of attitude by 48%, and decreases the odds of moderate or high practice by 59% when compared to Egypt as a graduation country.

(6) Rank of employment (dichotomized into beginner and senior)

Being a senior increases the odds of moderate or high knowledge by 19%, nonsignificantly increases the odds of having a moderate or high score in terms of attitude by 59%, and increases the odds of moderate or high practice by 3.73 compared with junior-level participants.

**Table 7:** Multiple ordinal regression to show the predictors of the ordinal 3 outcomes (knowledge, attitude, and practice).

	K			A			P		
Predictors	Odds Ratios	CI	*p*	Odds Ratios	CI	*p*	Odds Ratios	CI	*p*
Low|Moderate	0.69	0.15–3.19	0.637	0.20	0.01–4.75	0.319	0.55	0.11–2.71	0.460
Moderate|High	8.25	1.71–39.68	0.009	0.38	0.02–8.79	0.545	31.52	5.46–182.01	<0.001
Age	0.97	0.92–1.03	0.351	1.09	0.98–1.25	0.165	1.02	0.96–1.08	0.577
Gender [Female]	1.24	0.40–3.82	0.706	2.24	0.29–46.43	0.492	1.80	0.56–6.16	0.336
Experience	1.42	0.63–3.22	0.402	0.29	0.06–1.23	0.103	0.63	0.27–1.47	0.286
Last Degree [Higher]	3.56	1.48–8.86	0.006	5.04	1.12–28.48	0.047	1.58	0.66–3.84	0.311
Country Of Graduation [Others]	1.70	0.71–4.06	0.232	0.52	0.14–2.52	0.362	0.41	0.16–1.00	0.054
Country Of Graduation [Saudi Arabia]	0.47	0.16–1.31	0.158	0.29	0.07–1.31	0.091	0.58	0.21–1.57	0.289
Rank Of Employment [Senior]	1.19	0.45–3.06	0.721	1.59	0.28–29.96	0.665	3.73	1.19–12.85	0.031
Observations	209			209			209		
R^2^ Nagelkerke	0.079			0.074			0.080		

## 4. Discussion

The community pharmacists’ KAP about PV and ADRs is an important aspect of pharmaceutical care. To the best of our knowledge, this is the first study from the Qassim region to report KAP about PV among community pharmacists. Since pharmacists are frontline healthcare professionals dealing with patients on a daily basis, their role is critical in ADR identification, prevention, and reporting. Similarly, the evaluation of community pharmacists’ KAP about PV and their attitudes, practices, and barriers towards ADR reporting is critical to safeguarding public health.

The majority (62%) of participants correctly identified the WHO definition of PV, which is similar to other studies carried out in Kuwait (62%) [15], Sudan (52%) [17], Punjab, India (77%) [18], and Majmaah, Saudi Arabia (64%) [19]. In contrast, the correct definition of ADRs as per WHO was slightly lower (59%) in the current study as compared to the Kuwait study [15] (77.6%) but far better than Sudan, where only 8% could correctly identify the definition of ADR [17]. The reason behind the varying percentages between the studies may be due to the differences in sample size, study settings, duration, and population. Knowledge about ADR reporting centers in Saudi Arabia was 35% in our study, which is better compared to studies conducted in other parts of Saudi Arabia, such as Jizan (7%) [20], Riyadh (22%) [12], and the Alahsa region (10%) [21]. Interestingly, a recent study from Pakistan revealed that, among healthcare professionals, pharmacists had higher knowledge about PV [22]. This indicates that pharmacists are one of the most important members of the healthcare team to ensure medication safety during practice [23].

The Saudi healthcare system has a very well-structured ADR reporting system with a well-defined vision, mission, and goals. The mechanism for ADR reporting is in place, and it is regarded as a component of pharmacy and healthcare laws. The documentation of ADR is being updated and improved in all of the Ministry of Health (MOH) hospitals in Saudi Arabia [24]. Thus, pharmacists from the community or hospitals are in an ideal position to use these services to safeguard medication use. Although MOH and SFDA insist on ADR reporting by all community pharmacists, many of them are not performing this task due to a lack of awareness about the consequences of ADRs or due to negligence [12].

The pharmacist’s attitudes towards ADR reporting are vital in therapeutic decision making but are severely compromised due to inadequate or poor reporting [25]. The majority of participants in this study agreed on the importance of ADR reporting, corroborating that all ADRs should be reported, considering it their professional obligation, in agreeance with another study [17]. Moreover, the majority agreed with the notion that ADR reporting is necessary and will have a positive impact on the healthcare system. This clearly implies that community pharmacists have positive attitudes towards ADR reporting and want to promote rational drug use. Participants in this study reported conducting medication reviews of their patients and identifying ADRs. However, very few reported it to the ADR reporting centers. Being a primary point of contact, community pharmacists are in an ideal position to offer post-marketing data on drug products [25]. Surprisingly, not many of them were aware of reporting ADRs to drug companies. Furthermore, a study from Delhi, India, reported that only 60% of healthcare providers have taken a course regarding the reporting of ADRs, and the authors also revealed that pharmacists had the least knowledge about the importance of PV [26].

Key barriers to ADR reporting identified by community pharmacists were varied, with the majority not knowing how to report ADRs, and not knowing what information to report. These factors have previously been reported in Saudi Arabia, where the ADR reporting system was newly established [12,21,27]. The ADR documentation system was first established in the early 2000s, and the initial draft was revised between 2012 and 2015 by MOH, Saudi Arabia. Additionally, many private hospitals have their own systems for recording ADRs, either manually or electronically, with an extensive checklist. Previously, the MOH institutions used a manual system for documenting ADRs; however, this system was later electronically transformed using the Survey Monkey system [24]. Although a well-established system is now available for hospital pharmacists to use for ADR reporting, community pharmacies still lack an effective ADR reporting system. Another study from Saudi Arabia in 2009 revealed that a physician-centered healthcare system was practiced in Saudi Arabia, and pharmacists believed that physicians were responsible for reporting ADRs [28].

It is suggested that community pharmacists should adhere to the guidelines followed by health authorities and drug companies’ ADR reporting systems to encourage spontaneous ADR reporting [29]. Moreover, community pharmacists can make use of the SFDA website, which has an online form for reporting ADRs [11]. To overcome the barriers to reporting ADRs, it is the primary duty of the health authority to encourage pharmacists to actively perform ADR reporting through continuous education and training. A cross-sectional study from Saudi Arabia also recommended the importance of PV in their practice to ensure safe and effective therapy [30]. An improvement in information about ADRs and correspondence from the high level to the grass-roots level in the health sector will help in the appropriate usage of PV. Patients should be instructed to report any negative effects upon taking medication and not be forced to obtain information on their medications via the Internet. Legitimate identification, detailing, and reporting would assist with executing the PV for the advancement of society [31].

Our study had a few limitations. Firstly, we conducted our study only in the Qassim region; hence, the results cannot be generalized to the entire Kingdom of Saudi Arabia. Secondly, our study focused only on community pharmacists and was based on the fact that community pharmacists are the most easily accessible healthcare professionals for the general public to gain an opinion on their medication use. Consequently, the results of our study cannot be extrapolated as complete information about PV as four general hospitals are meeting the requirement of healthcare facilities in Qassim and more than 300 hospital pharmacists are working in these hospitals.

## 5. Conclusions

It is evident that, despite several barriers, community pharmacists demonstrate positive views on the importance of ADR reporting. It is, therefore, expected that more avenues of education and training can be offered specifically for community pharmacists to enhance ADR reporting culture. Although the SFDA currently provides a centralized ADR reporting system in Saudi Arabia, healthcare professionals, including community pharmacists, need to be motivated and encouraged to report ADRs. Furthermore, community and hospital pharmacists can work together on medication reviewing and ADR reporting.

## Figures and Tables

**Table 1:** Demographic details of study participants (n = 209).

Demographic Characteristics	n (%)
**Age (Median, IQR)**	32 (27, 37)
**Gender**	
Male	193 (92%)
Female	17 (8%)
**Nationality**	
Egyptian	130 (62%)
Saudi	54 (26%)
Sudanese	10 (5%)
Yamen	9 (4%)
India	2 (1%)
Bangladesh	2 (1%)
Iraq	1 (0.5%)
Jordan	1 (0.5%)
**Rank of Employment**	
Pharmacist	184 (88%)
Senior Pharmacist	14 (7%)
Beginner Pharmacist	8 (4%)
Head of Pharmacy Specialist	2 (1%)
Senior Pharmacy Specialist	1 (0.5%)
Pharmacy Specialist	1 (0.5%)
**Qualification**	
Bachelor	152 (72%)
Pharm D	50 (24%)
Master	6 (3%)
PhD	2 (1%)
**Practice Years (Median, IQR)**	7.0 (3.0, 14.0)
**Location of Pharmacy**	
Unaizah	72 (34%)
Buraidah	52 (25%)
Alrass	40 (19%)
Bukairyah	18 (9%)
Badaie	14 (7%)
Riyadh Al Khabra	9 (4%)
Mithnab	4 (2%)

IQR: interquartile range.

**Table 2:** Knowledge about PV and ADRs (n = 209).

Knowledge Questions	n (%)
**The best definition of PV**	
The science and activities of detecting, assessing, understanding & preventing adverse effects (correct)	130 (62%)
The science of detecting the type & incidence of ADRs after a drug is marketed.	37 (18%)
The process of improving the safety of drugs	24 (11%)
I don’t know	13 (6%)
The science of monitoring ADRs happening in a Hospital	5 (2%)
**The purpose of PV**	
To enhance patients’ safety (correct)	144 (69%)
To identify unrecognized ADRs	19 (9%)
To identify predisposing factors to ADRs	18 (9%)
To calculate incidence of ADRs	16 (8%)
I don’t know	12 (6%)
**ADRs definition**	
Any noxious or undesired effect of a drug (correct)	123 (59%)
Adverse health outcomes	39 (19%)
Harm resulting from the use	20 (10%)
Other health problems	11 (5%)
Harm caused by drug overdose	9 (4%)
Adverse outcomes associated with drug	7 (3%)
**Common cause of ADR**	
Allergic reactions	143 (68%)
Drug interaction	126 (60%)
Undesirable effects	96 (46%)
Unsafe drug for the patient fast	67 (32%)
Incorrect administration	59 (28%)
Dosage increased or decreased too fast	44 (21%)
**Which ADRs should be reported**	
All of the above (correct)	131 (62%)
All serious	56 (27%)
ADRs to new drugs	9 (4.3%)
ADRs to herbal	5 (2.4%)
ADRs to vaccines	3 (1.4%)
Unknown ADRs to old drugs	3 (1.4%)
None of the above	2 (1%)
**Are ADRs classified as DTP?**	
Yes	142 (68%)
No	36 (17%)
I don’t know	31 (15%)
**Who is qualified to report ADRs?**	
Pharmacists	182 (87%)
Doctors	158 (76%)
Nurses	74 (35%)
Dentists	67 (32%)
Patients	56 (27%)
Physiotherapists	37 (18%)
**Organizations that educate HC professionals on safe medication practices**	
Canadian Patient Safety Institute	120 (58%)
International Medication Safety Network	92 (44%)
Institute for Safe Medication Practices	70 (33%)
**Are you aware of centers for reporting ADR in SA?**	
No	84 (40%)
Yes	73 (35%)
I don’t know	52 (25%)
**Where to report ADRs**	
I don’t know	149 (71%)
SFDA	36 (17%)
MOH	12 (6%)
WHO	2 (1%)
**The most preferable method to report ADR**	
Email/on Website	118 (57%)
Direct contact	56 (27%)
Telephone	23 (11%)
Post	8 (4%)
Other (please specify)	4 (2%)

ADR: adverse drug reactions, DTP: drug therapy problems, HC: healthcare, MOH: Ministry of Health, PV: pharmacovigilance, SA: Saudi Arabia, SFDA: Saudi Food and Drug Authority, WHO: World Health Organization.

**Table 3:** Attitude towards PV and ADRs reporting (n = 209).

Attitude Questions	Yes [n (%)]	No [n (%)]	IDK n (%)
Is it necessary to report ADR?	195 (93%)	7 (3%)	8 (4%)
Reporting ADR will positively impact HC system	196 (94%)	5 (2%)	9 (4%)
Should ADR reporting and PV should be reported in HC setting?	183 (88%)	12 (6%)	15 (7%)
Is ADR reporting a professional obligation?	172 (82%)	20 (10%)	18 (9%)
Conducting a medication review can prevent ADR	167 (80%)	17 (8%)	26 (12%)
Are you willing to implement ADR reporting in your practice?	155 (74%)	33 (16%)	22 (10%)

ADR: adverse drug reactions, IDK: I don’t know, HC: healthcare. Total percentage may not be 100% due to the choice given for multiple responses.

**Table 4:** Practice towards PV and ADRs reporting.

Practice Questions	Yes n (%)	No n (%)	IDK n (%)
Have you ever conducted a medication review with your patients?	142 (68%)	68 (33%)	
Have you ever identified an ADR in any patient?	114 (55%)	96 (46%)	
Have you ever reported an ADR?	46 (22%)	162 (77%)	2 (1)%
**Do you know to whom ADR should be reported?**			
Saudi Food and Drug Authority (SFDA)	137 (66%)	72 (34%)	
The ministry of health(MOH)	102 (49%)	107 (51%)	
Drug company	43(21%)	166(79 %)	

ADR: adverse drug reactions, IDK: I don’t know. Total percentage may not be 100% due to the choice given for multiple responses.

**Table 5:** Barriers towards PV and ADRs reporting.

What Factors Do You Think May Be Discouraging of Reporting ADRs?	Yes
Lack of formal process	131 (63%)
Lack of knowledge by the patients about their medications	119 (57%)
Lack of time	86 (41%)
Lack of training on how to conduct a medication review	84 (40%)
Communication (language barrier) difficulties with the patients	56 (27%)
Others	17 (8%)
Total percentage may not be 100% due to the choice given for multiple responses	

**Table 6:** Factors discouraging PV and ADRs reporting.

**What Factors Do You Think May Be Discouraging of Reporting ADRs?**	**Yes**	**No**
Not knowing how to report	123 (59%)	87 (41%)
Not knowing what information to report	70 (33%)	140 (67%)
Thinking it is not important to report an ADR incident	55 (26%)	155 (74%)
Managing patients is more important than reporting ADR	51 (24%)	159 (76%)
Patient confidentiality issues	37 (18%)	173 (82%)
It is not part of my job to report ADRs	25 (12%)	185 (88%)
**What do you think are the barriers for Saudi Arabia to have a formal PV Center?**	**Yes**	**No**
None	166 (79%)	
Lack of training	23 (11%)	
Lack of staff	13 (6%)	
Workload	6 (3%)	
Lack of time	2 (1%)	
Total percentage may not be 100% due to the choice given for multiple responses		

## Data Availability

Data will be made available on a reasonable request.

## Supplementary Materials

The following supporting information can be downloaded at: https://www.mdpi.com/article/10.3390/ijerph20043548/s1.
