# Supplementary material for: Evaluation of Knowledge, Attitudes, and Practices about Pharmacovigilance among Community Pharmacists in Qassim, Saudi Arabia
Source: Int J Environ Res Public Health. 2023 Feb 17;20(4):3548. doi: 10.3390/ijerph20043548 (PMC9958751; doi:10.3390/ijerph20043548)
Supplement: Supplementary file 1 [file ijerph-20-03548-s001.zip › ijerph-2136676-supplementary.pdf]

Pharmacists in Qassim, Saudi Arabia. **Survey**

Instructions: We request your time and cooperation in completing this survey.

**Knowledge and awareness of pharmacovigilance (PV) and adverse drug reactions (ADRs)**	
**1. Which of the following BEST defines PV (WHO* definition)?**<br><br>**(Please select ONE answer only)**<br><br>*WHO = World Health Organization	1 □ The science of monitoring ADRs happening in a Hospital.<br>2 □ The process of improving the safety of drugs<br>3 □ The science and activities of detecting, assessing, understanding & preventing adverse effects<br>4 □ The science of detecting the type & incidence of ADRs after a drug is marketed.<br>5 □ I do not know
**2. The purpose of PV is:**<br><br>**(Please select ONE answer only)**	1 □ To enhance patients' safety in relation to use of drugs<br>2 □ To calculate incidence of ADRs<br>3 □ To identify predisposing factors to ADRs<br>4 □ To identify unrecognised ADRs<br>5 □ I do not know
**1. Which of the following defines ADRs correctly (WHO definition)?**<br><br>**(Please select ONE answer only)**	1 □ Adverse health outcomes associated with inappropriate drug use<br>2 □ Harm caused by drug overdose<br>3 □ Harm resulting from the use of substandard/counterfeit drugs<br>4 □ Any noxious or undesired effect of a drug occurring at normal doses, during normal use<br>5 □ Adverse outcomes associated with drug impurity<br>6 □ Other health problems associated with drug use
**2. Which of the following could be a common cause of ADRs?**<br>***(You may tick MORE THAN ONE)***	1 □ Undesirable effect 2 □ Allergic reaction<br>3 □ Incorrect administration 4 □ Drug interaction<br>5 □ Unsafe drug for the patient 6 □ Dosage increased or decreased too fast<br>7 □ Other (please specify) …………………………………………
**3. In your opinion, which ADRs should be reported?**<br><br>**(Please select ONE answer only)**	1 □ All serious ADRs 2 □ ADRs to herbal and non-allopathic drugs<br>3 □ ADRs to new drugs 4 □ ADRs to vaccines

	5 □ Unknown ADRs to old drugs 6 □ All of the above<br>7 □ None of the above
**4. Are ADRs classified as drug therapy problems (DTP)?**	1 □ Yes 2 □ No 3 □ I do not know
**5. In your opinion, which of these are qualified to report ADRs?**<br>***(You may tick MORE THAN ONE)***	1 □ Doctors 2 □ Nurses 3 □ Pharmacists<br>4 □ Dentists 5 □ Physiotherapists 6 □ Patients<br>7 □ Other (please specify) ………………………………………………
**6. Have you heard of the following medication safety processes?**<br>***(You may tick MORE THAN ONE)***	1 □ Medication reconciliation<br>2 □ Medication check review<br>3 □ Medication history<br>4 □ I don't know
**7. Are you aware of organizations that educate healthcare professionals on safe medication practices?**<br>***(You may tick MORE THAN ONE)***	1 □ Institute For Safe Medication Practices<br>2 □ International Medication Safety Network<br>3 □ Canadian Patient Safety Institute<br>4 □ World Health Organization (WHO)<br>5 □ Other: ______________________________<br>6 □ I don't know
**8. Are you aware of any Center or ADR reporting system in Saudi Arabia?**	1 □ Yes 2 □ No 3 □ I do not know<br>**If YES, where?** ………………………………………………
**9. Which method would you prefer to send ADR information to an ADR Reporting Center?**<br>**(Please select ONE answer only)**	1 □ Direct contact 2 □ Post 3 □ Telephone<br>4 □ Email/on Website 5 □ Other (please specify) …………………………
**Perception and attitude towards PV and ADRs reporting**	
**10. In your opinion, do you think it is necessary to report ADRs?**	1 □ Yes 2 □ No 3 □ I do not know
**11. In your opinion, is ADR reporting a professional obligation?**	1 □ Yes 2 □ No 3 □ I do not know
**12. Do you think that ADR reporting will affect healthcare system in a positive way?**	1 □ Yes 2 □ No 3 □ I do not know

**13. In your opinion, do you think that conducting a medication review can prevent ADRs?**	1 □ Yes 2 □ No 3 □ I do not know
**14. Should ADR reporting and PV be taught in detail to healthcare professionals?**	1 □ Yes 2 □ No 3 □ I do not know
**15. Are you willing to implement ADR reporting in your practice?**	1 □ Yes 2 □ No 3 □ I do not know
**Practices and barriers of ADR identification and reporting**	
**16. Have you ever conducted a medication review with your patients?**	1 □ Yes 2 □ No<br>**If YES, <u>at which frequency</u>? (Please choose ONE):**<br>1 □ rarely 2 □ occasionally 3 □ often 4 □ always
**17. What are the barriers to conducting a medication review?**<br>***(You may tick MORE THAN ONE)***	1 □ Lack of time<br>2 □ Lack of training on how to conduct a medication review<br>3 □ Lack of a formal process in place<br>4 □ Communication (language barrier) difficulties with the patients<br>5 □ Lack of knowledge by the patients about their medications<br>6 □ Other (please specify) ....
**18. Have you ever identified an ADR in any patient?**	1 □ Yes 2 □ No<br>**If YES, <u>how many</u> till date? (Please choose ONE):**<br>1 □ <5 times 2 □ 5-10 times 3 □ >10 times
**19. Have you ever reported an ADR?**	1 □ Yes 2 □ No<br>**If YES, <u>on what medications</u>? ....**
**20. Do you know to whom ADR should be reported?**<br>***(You may tick MORE THAN ONE)***	1 □ The Ministry of health (MOH)<br>2 □ Saudi Drug and Food Control Administration<br>3 □ Drug Company<br>4 □ My hospital or clinic<br>5 □ Other (please specify) ....<br>6 □ I do not know
**21. What factors do you think may be discouraging of reporting ADRs?**<br>***(You may tick MORE THAN ONE)***	1 □ Not knowing how to report<br>2 □ Not knowing what information to report<br>3 □ Thinking it is not important to report an ADR incident<br>4 □ Managing patients is more important than reporting ADR<br>5 □ It is not part of my job to report ADRs

	6 □ Patient confidentiality issues<br>7 □ Other (please specify) …………………………………………
**22. Are ADRs being reported as part of "incident reports" in your institution?**	1 □ Yes 2 □ No 3 □ I do not know<br>**If YES, <u>what do you do with these reports</u>?**<br>……………………………………………………………………………………………………

23. What do you think are the barriers for Saudi Arabia to have a formal PV Center (as opposed to the Gulf Countries)?

………………………………… ……………………………………… ………………………………………

24. Any suggestions/comments you would like to provide?

………………………………… ……………………………………… ………………………………………

Demographics

25. Age ……….years

26. Gender 1 □ Male 2 □ Female

27. Nationality 1 □ Saudi 2 □ Non-Saudi **(Please specify)**………………………………..

28. What is your rank of employment? (Please choose ONE)

1 □ **Beginner Pharmacist (صيدلي مبتدئ)** 2 □ **Pharmacist (صيدلي)**
3 □ **Senior Pharmacist (صيدلي أول)** 4 □ **Pharmacy Specialist (اختصاصي)**
5 □ **Senior Pharmacy Specialist (اختصاصي أول صيدلة)** 6 □ **Head of Pharmacy Specialist (رئيس اختصاصيين)**

What is your <u>LAST</u> degree in pharmacy?
1□ **Bachelor** 2 □ **Pharm D** 3 □ **Master** 4 □ **PhD** 5 □ **Other** …………………………………

29. Country of graduation: …………………………………………

30. Practice years ……………………………………………(years)

31. Location:……………………………………………..

Thank you for your cooperation
